# SAR1A Induces Cell Growth and Epithelial–Mesenchymal Transition Through the PI3K/AKT/mTOR Pathway in Head and Neck Squamous Cell Carcinoma: An In Vitro and In Vivo Study

**DOI:** 10.3390/biomedicines12112477

**Published:** 2024-10-28

**Authors:** Shizhen Fang, Jie Wang, Tianyi Liu, Yang Jiang, Qingquan Hua

**Affiliations:** 1 Department of Otolaryngology-Head and Neck Surgery, Renmin Hospital of Wuhan University, 238 Jie-Fang Road, Wuhan 430060, China; 2022203020011@whu.edu.cn (S.F.); 2013302180159@whu.edu.cn (J.W.); rm003860@whu.edu.cn (T.L.); 2 Central Laboratory, Renmin Hospital of Wuhan University, 238 Jie-Fang Road, Wuhan 430060, China

**Keywords:** SAR1A, head and neck squamous cell carcinoma, PI3K/AKT/mTOR, LY294002

## Abstract

Objectives: Head and neck squamous cell carcinoma (HNSCC) ranks sixth globally, with a 50% five-year survival rate. SAR1A exhibits high expression levels in various tumor types, yet its specific role in HNSCC remains to be clarified. Methods: In vitro assays, such as CCK8, EdU, colony formation, wound-healing, transwell, and Western blotting analyses, as well as in vivo assays, such as tumor xenografts and lung metastasis models, were conducted to evaluate the impacts of SAR1A on HNSCC proliferation, migration, and invasion. Transcriptome sequencing and KEGG enrichment pathway analysis revealed evident alterations in the PI3K/AKT/mTOR(PAM) pathways. LY294002 (a PI3K/AKT inhibitor) was used to investigate the role of the PAM pathway in proliferation, migration, and invasion in HNSCC. Results: Univariate and multivariate Cox regression were conducted to screen SAR1A as a gene prognostic biomarker in HNSCC, and it was validated in the Cancer Genome Atlas (TCGA) database. Functional assays demonstrated that the depletion of SAR1A leads to suppressed proliferation, migration, and invasion of HNSCC cells. This is accompanied by a decrease in the expression of epithelial–mesenchymal transition (EMT)-related markers in HNSCC cell lines. In addition, the diminished capacities of proliferation, migration, and invasion observed in SAR1A knockdown cells were reversed upon the overexpression of SAR1A. Furthermore, RNA-seq and KEGG enrichment analysis demonstrated a significant alteration in the PAM pathway following SAR1A knockdown. LY294002 effectively mitigated the increased proliferation, migration, and invasion induced by SAR1A overexpression. Conclusions: SAR1A facilitates HNSCC proliferation and EMT via the PI3K/AKT/mTOR pathway.

## 1. Introduction

Head and neck squamous cell carcinoma (HNSCC), ranks sixth globally and continues to show a growing incidence and is projected to increase by 30% by 2030 [1]. The current primary approach for treating HNSCC involves surgical intervention, chemotherapy, radiotherapy, and molecular-targeted therapy [2]. While some premalignant oral lesions have the potential to progress into invasive cancer, a considerable number of patients are initially found with advanced-stage HNSCC [1]. The five-year survival rate for HNSCC is 50% [3]. Therefore, it is imperative and essential to discover new molecular targets for regulating the proliferation and progression of HNSCC.

The PI3K/AKT/mTOR (PAM) pathway is highly conserved in eukaryotic cells and is involved in promoting cell growth, survival, and the cell cycle [4]. Approximately 50% of tumors exhibit aberrations in the PAM pathway [5]. The PAM pathway is the most commonly altered pathway in HNSCC [1]. Dickkopf-related protein 3 (DKK3) contributes to the malignant phenotype through the PAM and MAPK signaling pathways in HNSCC [6].

SAR1A (secretion-associated Ras-related GTPase 1A), a member of the small GTPase superfamily, mediates the transportation of vesicles from the Golgi to the endoplasmic reticulum (ER). Mammals possess two paralogues of Sar1 in Homo sapiens—specifically, SAR1A and SAR1B [7]. SAR1A is involved in various physiological and pathological processes, including neurological disorders [8], metabolic diseases [9], circulatory function [10], muscle development [11], hematological disorders [12,13], and multiple cancers [14,15,16]. For example, in neurological diseases, SAR1A levels are significantly reduced in Alzheimer’s disease (AD) [17]. In metabolic diseases, SAR1A mutations impair insulin processing, leading to ER stress [18]. In the circulatory system, both SAR1A and SAR1B play crucial roles in the transport of the cardiac sodium channel Na_v_1.5 and the generation of cardiac sodium current (I_Na_) [10]. In hypertrophic scarring fibroblasts (HTSFs), SAR1A expression is upregulated, serving as a positive regulator for PC-I secretion via TGF-β1-mediated TAK1-JNK and TAK1-p38 pathways [19]. In malignant tumors, SAR1A exhibits complex roles, such as promoting osteosarcoma metastasis through the RhoA/YAP pathway [20] and inhibiting ERK signaling, thereby suppressing the growth of hepatocellular carcinoma (HCC) cells [21]. While previous studies have explored the role of SAR1A in various cancers, its specific implications in HNSCC have remained unclear.

In summary, this study utilizes bioinformatics analysis to demonstrate the high expression of SAR1A in HNSCC and its association with poor prognosis. Transcriptome sequencing uncovers the PAM pathway and serves as the downstream of SAR1A in HNSCC. In addition, both in vivo and in vitro experiments confirm that SAR1A promotes the proliferation, migration, and invasion of HNSCC cells through the PAM pathway.

Our study identifies SAR1A as a promising biomarker and highlights its significance in HNSCC progression. Additionally, it reveals the PAM pathway as a novel mechanism of action, providing valuable insights into potential therapeutic targets.

## 2. Materials and Method

### 2.1. Data Acquisition

RNA-seq datasets were obtained from the TCGA database https://cancergenome.nih.gov/ (accessed on 30 June 2022) and analyzed using the R package “TCGA-Assembler”. A total of 546 HNSCC patients from the TCGA database (44 normal, 502 with tumors) were included in our study as the training dataset.

### 2.2. Selection of Autophagy-Associated Genes in HNSCC

Autophagy-associated genes were downloaded from the Human Autophagy Database (HADb) library http://www.autophagy.lu/ (accessed on 30 June 2022). The Limma package in the R language (v3.34.7) was used to screen autophagy-associated DEGs in HNSCC.

### 2.3. Construction of a Risk Model in HNSCC

A univariate Cox regression was conducted using the “survival” package in R. Multivariate Cox regression was conducted on significant genes (*p* < 0.05) identified from the univariate regression. The risk score function was defined and plotted accordingly.

$$\mathrm{Risk}\;\mathrm{score}=\sum_{n=1}^{K}T_{i}\times C_{i}$$

Here, *T* represents the expression level of individual genes, *C* denotes their corresponding multivariate regression coefficients, and *K* signifies the quantity of these genes present in each patient.

### 2.4. HNSCC Tissue Microarray

Forty-three HNSCC tissue and adjacent tissue microarrays were procured from WhuIwill Biological Technology Co., Ltd. (Wuhan, China) (IWLT-N-90LS51) for investigating SAR1A expression through immunohistochemical (IHC) staining.

### 2.5. Analysis of Gene Expression of Interest

UALCAN http://ualcan.path.uab.edu (accessed on 6 August 2024) was employed for analyzing gene expression across various cancer types by utilizing data sourced from TCGA. We conducted an investigation of the expression of SAR1A in 29 cancer types, and visual representations such as graphs and plots are provided to illustrate the expression profiles.

### 2.6. Overall Survival Analysis

The Kaplan–Meier Plotter (KM Plotter) (Jerusalem, Israel) was employed to evaluate the association between gene expression and overall survival (OS). The hazard ratio (HR) was calculated with the 95% confidence interval (CI) and log-rank *p* value.

### 2.7. Analysis of DNA Methylation

The SMART http://www.bioinfo-zs.com/smartapp (accessed on 6 August 2024) was utilized to investigate the methylation of SAR1A in HNSCC. A SMART summary was conducted to visually depict the chromosomal distribution. Furthermore, a pan-cancer analysis of gene expression was performed to assess the epigenetic regulation of SAR1A in various cancers by utilizing parameters such as the median and M-value.

### 2.8. TIMER2.0 Analysis

TIMER2.0 http://timer.cistrome.org/ (accessed on 6 August 2024)was utilized to assess the correlation between SAR1A expression level and its clinical relevance to immune subsets. Furthermore, the impact of the target gene on immune cell purity, as well as specific subpopulations such as T cells, B cells, dendritic cells, neutrophils, and macrophages, was investigated.

### 2.9. Cell Culture and Treatment

Cal-27 (#STCC12901P, Servicebio, Wuhan, China) cells, 293T (#STCC10301P, Servicebio, Wuhan, China) cells, and Tu686 (Hunan Fenghui Biotechnology, Changsha, China) were obtained and cultured in Dulbecco’s modified Eagle’s medium (#G4511, Servicebio, Wuhan, China) supplemented with 10% fetal bovine serum (#164210-50, Procell, Wuhan, China) and 1% penicillin–streptomycin (#G4016, Servicebio, Wuhan, China). Cells were treated using the PI3K/AKT inhibitor LY294002 (Cat. HY-10108; MedChemExpress, Monmouth Junction, NJ, USA) at 25 μM for 24 h [22].

### 2.10. Construction of the SAR1A Lentivirus

The overexpression group utilized an empty vector control, while the knockdown group employed a scrambled shRNA control. The plasmids PMD2.0G, SPAX2, and the target plasmids (SAR1A-OE, vector, SAR1A-sh1, SAR1A-sh2, or scramble) were co-transfected into 293T cells at 37 °C. After 48 and 72 h, the culture supernatant was collected, concentrated, and purified. The cells were then seeded onto a 6-well plate and transfected with the lentivirus for 24 h at 37 °C. Following this, the transfection solution was replaced with a complete medium, and stable strain selection was performed after 96 h of culture with puromycin at 37 °C. The puromycin concentration used for selection was 2.5 µg/mL. Western blot analysis was conducted to evaluate the success of the transfection by detecting protein expression.

### 2.11. Real-Time Quantitative PCR

Total RNA was extracted utilizing an RNA Kit (Aidlab Biotech, Beijing, China). Then, cDNA was synthesized using a cDNA Synthesis Kit (#G3333, Servicebio, Wuhan, China). Real-time quantitative PCR was conducted using 2×Universal Blue SYBR Green qPCR Master Mix (#G3328, Servicebio, Wuhan, China) using a qPCR machine (Roche LightCycler480II, Basel, Switzerland). The relative mRNA expression level was normalized to GAPDH and analyzed by utilizing the 2^−∆∆Ct^ method. All samples were assayed in triplicate. The primer sequences are presented in Supplementary Table S1.

### 2.12. CCK-8 Assay

A total of 1000 cells per well in 96-well plates were incubated for 24, 48, 72, and 96 h. The cells were incubated with 10% CCK8 reagent (Servicebio, Wuhan, China) for one hour, followed by measurement of the absorbance at 450 nm utilizing a microplate reader (Perkin Elmer, Waltham, MA, USA).

### 2.13. Colony Formation

A total of 1000 cells per well were seeded for 14 days. After fixation with 4% paraformaldehyde (#G1101, Servicebio, Wuhan, China), the plates were dyed with a 0.5% crystal violet solution (BL802A, Biosharp Life Sciences, Beijing, China). Following plate staining, colonies were imaged and examined with ImageJ software (v1.46r) (WS Rasband, ImageJ, NIH, Bethesda, MD, USA).

### 2.14. 5-Ethynyl-20-Deoxyuridine (EdU) Assay

The BeyoClick™ EdU Kit (Beyotime, Shanghai, China) was used. Cells were treated with EdU for 2 h, fixed with 4% paraformaldehyde (#G1101, Servicebio, Wuhan, China), and permeabilized with 0.1% Triton X-100 (#GC204003, Servicebio, Wuhan, China). Nuclei were stained with DAPI. Cellular images were taken with a fluorescence microscope (Olympus BX53, Tokyo, Japan).

### 2.15. Wound-Healing Assay

The cells were cultured and incubated at 37 °C in a medium containing 10% FBS until they reached 80% to 90% confluence. A scratch was generated using a 10 µL pipettor in cells at 80–90% confluence. The cells were then incubated in a serum-free medium for 0 and 24 h, and images of the scratch were captured using a microscope (Olympus IX71, Tokyo, Japan).

### 2.16. Transwell Assay

Transwell chambers (Corning Incorporated, Corning, NY, USA) were precoated with or without Matrigel to evaluate the invasive and migratory capabilities of the cells. While the bottom chambers were filled with a medium containing 20% FBS, the upper chambers of the cells were treated with a serum-free medium at 37 °C for 48 h. Afterward, the cells were fixed, stained, and photographed under a microscope (Olympus BX53, Tokyo, Japan).

### 2.17. RNA-Seq Data Analysis

Cal-27 cells from SAR1A knockdown and control groups were sent to IGENBOOK Biotechnology Company (Wuhan, China) for high-throughput transcriptome sequencing using the Illumina NovaSeq platform. The sequence alignment results for each sample group were filtered to identify DEGs that exhibited significant changes following treatment. This was accomplished using the R package edgeR, applying criteria of |log2FC| ≥ 1 and *p* < 0.05. The results were utilized in the KEGG database for additional enrichment analysis.

### 2.18. Xenograft Model

In total, 30 female BALB/c nude mice, aged five weeks, were randomly allocated to six groups (*n* = 5): scramble (injected with scramble cells), SAR1A-sh1 (injected with SAR1A-sh1 cells), SAR1A-sh2 (injected with SAR1A-sh2 cells), vector (injected with vector cells), SAR1A-OE (injected with SAR1A-OE cells), and SAR1A-OE + LY294002 (injected with SAR1A-OE cells). The corresponding groups of cells were digested and suspended in PBS to a density of 1 × 10^7^ cells/mL. A volume of 200 μL (equivalent to 5 × 10^6^ cells) of this cell suspension was subcutaneously injected into the dorsal region of nude mice. One week later, the xenograft mice in the SAR1A-OE + LY294002 group were administered LY294002 via intraperitoneal injection at a dosage of 100 mg/kg three times per week for a duration of 2 weeks [23,24]. The other two groups (vector and SAR1A + OE) were given normal saline intraperitoneally. Tumor dimensions were assessed at three-day intervals, and tumor volume was determined using the following formula: V (mm^3^) = (width)^2^ × length/2. After 28 days, nude mice were euthanized via cervical dislocation following anesthesia with isoflurane. The tumors were excised, weighed, and fixed with 4% neutral paraformaldehyde. In the xenograft tumor model, the innovative application of the PI3K/AKT inhibitor LY294002 more comprehensively validated the role of SAR1A in promoting HNSCC cell proliferation via the PI3K/AKT/mTOR pathway.

### 2.19. In Vivo Metastasis Assays and Live Imaging with Luciferase

For the tumor metastasis assay, 30 female BALB/c nude mice that were aged five weeks were randomly allocated to six groups (*n* = 5): the scramble group, the SAR1A-sh1 group, the SAR1A-sh2 group, the vector group, the SAR1A-OE group, and the SAR1A-OE + LY294002 group. The corresponding groups of cells were transduced with lentivirus expressing luciferase. Cells were collected and suspended in PBS at a density of 1 × 10^7^ cells/mL. A 200 μL volume of the suspension was injected via the tail vein. One week later, the SAR1A-OE + LY294002 group was administered LY294002 via intraperitoneal injection at a dosage of 100 mg/kg three times per week for a duration of 2 weeks. The other two groups (SAR1A-EV and SAR1A + OE) were given normal saline intraperitoneally. After a 28-day period, D-luciferin potassium salt (ST196, Beyotime, Shanghai, China) was dissolved in D-PBS (G4200, Servicebio, Wuhan, China) at a concentration of 15 mg/mL and administered intraperitoneally at a dose of 10 μL/g body weight, following isoflurane anesthesia in the mice. Image acquisition was performed using the IVIS Lumina XRMS Series III (PerkinElmer, Waltham, MA, USA). All lung and liver specimens were excised and fixed in 4% neutral paraformaldehyde. The tail vein lung metastasis model offers a more precise simulation of the process by which tumor cells migrate to the lungs through the bloodstream and form metastatic lesions, enabling the assessment of their metastatic and invasive potential. Furthermore, the innovative utilization of LY294002 in vivo in mice provided stronger evidence for SAR1A’s promotion of HNSCC cell metastasis and invasion via the PI3K/AKT/mTOR pathway.

### 2.20. Western Blotting Analysis

The proteins were separated by SDS-PAGE at a constant voltage and subsequently transferred to PVDF membranes (Millipore, Burlington, NJ, USA) using a constant current. Following membrane blocking for one hour, they were incubated at 4 °C for 12–16 h with corresponding antibodies: rabbit anti-GAPDH (1:1000, GB10002, Servicebio, Wuhan, China); rabbit anti-SAR1A (1:1000, A7476, ABclonal, Wuhan, China); mouse anti-E-cadherin (1:2000, 60335-1-Ig, Proteintech, Wuhan, China); mouse anti-N-cadherin (1:5000, 66219-1-Ig, Proteintech, Wuhan, China); rabbit anti-Vimentin (1:2000, A19607, ABclonal, Wuhan, China); rabbit anti-MMP2 (1:1000, GB11130-100, Servicebio, Wuhan, China); rabbit anti-p-PI3K (1:1000, 4228P, Cell Signaling Technology, Danvers, MA, USA); rabbit PI3K (1:1000, AF6241, Affinity Biosciences, Changzhou, China); rabbit p-AKT (1:1000, T40067, Abmart, Shanghai, China); rabbit AKT (1:1000, 4691P, Cell Signaling Technology, Danvers, MA, USA); rabbit p-mTOR (1:1000, T56571, Abmart, Shanghai, China); and rabbit mTOR (1:1000, AF6308, Affinity Biosciences, Changzhou, China). They were incubated for 1 h at room temperature with secondary antibodies, namely anti-mouse IgG, HRP-linked antibody (1:8000, 7076P2, Cell Signaling Technology, Danvers, MA, USA) and HRP-conjugated goat anti-rabbit IgG(H+L) (1:10,000, SA00001-2, Proteintech, Wuhan, China). The immunoreactive protein bands were visualized using chemiluminescence (#BL520A, Biosharp Life Sciences, Beijing, China) using an ECL imager (ChemiDoc, Bio-Rad, Hercules, CA, USA).

### 2.21. Statistical Analysis

The experiments were conducted in triplicate, and the statistical analysis of the data was performed using GraphPad Prism software v10.0 (GraphPad Software Inc., La Jolla, CA, USA). Quantitative data are displayed as the mean ± SD. Two groups were compared using a two-tailed unpaired Student *t*-test, while three or more groups were compared using a one-way analysis of variance (ANOVA). A *p* value below 0.05 was deemed to be statistically significant. For each experiment, the significance levels are indicated as follows: * denotes *p* < 0.05, ** denotes *p* < 0.01, and *** denotes *p* < 0.001.

## 3. Results

### 3.1. The Upregulation of SAR1A in HNSCC Samples

**Figure 1:**
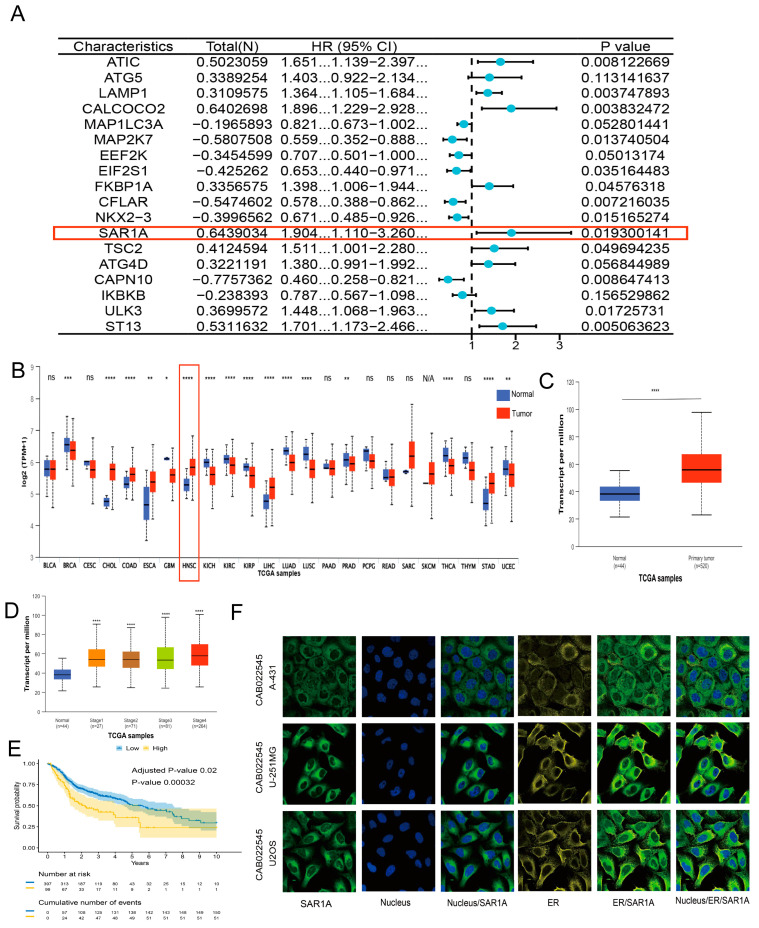
The upregulation of SAR1A in HNSCC samples: (**A**) identification of prognosis-associated ARGs using multivariate Cox analysis, SAR1A marked with a red box; (**B**) UALCAN analysis of SAR1A expression in 24 cancers from the TCGA database; normal tissues are depicted in blue and tumor tissues are depicted in red, HNSC marked with a red box; (**C**) SAR1A expression in HNSCC based on normal samples and primary tumor samples; (**D**) expression of SAR1A in HNSCC based on normal samples and samples at different cancer stages; (**E**) Kaplan–Meier curve based on the TCGA-HNSC database (https://tau.cmmt.ubc.ca/cSurvival/) (accessed on 7 October 2024) showing the survival probabilities of HNSCC patients at ten years based on high and low SAR1A expression levels; (**F**) the localization of SAR1A was detected in A-431, U-2OS, and U-251 MG cells using immunofluorescence staining from the HPA database. N/A, not applicable; * *p* < 0.05; ** *p* < 0.01; *** *p* < 0.001; **** *p* < 0.0001; ns, not significant.

To explore the expression of ARGs in HNSCC, 502 HNSCC and 44 normal samples were downloaded from the TCGA, and 232 ARGs were obtained from the HADb http://www.autophagy.lu/ (accessed on 6 August 2024). As depicted in Supplementary Figure S1, the expression levels of 38 ARGs showed significant differences between normal and HNSCC samples (*p* < 0.05, fold change > 1), with 11 genes being downregulated and 27 genes being upregulated. We conducted univariate Cox regression to assess the hazard ratio (HR) of 232 ARGs in HNSCC using data from the TCGA database in order to investigate their potential association with the prognosis of HNSCC. The forest plot (Supplementary Figure S2) indicated a significant correlation between the high expression of 22 genes and poor OS in HNSCC patients. Conversely, the elevated expression of 26 genes was correlated with improved OS. We conducted multivariate Cox regression analysis (Figure 1A) and constructed a risk model, resulting in the calculation of risk scores. Among the ARGs, 13 showed *p* values less than 0.05, with 8 genes exhibiting elevated expression levels that correlated with poorer OS. Five genes were linked to better OS.

Based on the risk scores generated by the risk model, we categorized all patients into low-risk and high-risk groups using the median risk score and observed an increase in mortality as the patients’ risk scores increased (Supplementary Figure S3A,B). The Kaplan–Meier curves showed that the median survival time was shorter in the high-risk group (Supplementary Figure S3C). The uniforest and multiforest plots demonstrated an association between the risk score and OS (Supplementary Figure S3D). ROC curves were created to assess the efficacy of Cox regression, with an AUC of 0.744 indicating accurate prognostic power for the risk score (Supplementary Figure S3E).

SAR1A exhibited the highest HR in ARGs. So, we assessed the expression of the SAR1A gene in 24 different types of cancer, including HNSCC https://ualcan.path.uab.edu/ (accessed on 6 August 2024). Figure 1B illustrates the upregulation of the SAR1A gene in six cancer types and the downregulation of the SAR1A gene in nine cancer types in comparison with normal tissues. To investigate SAR1A expression in HNSCC patients specifically, we analyzed 44 normal samples and 520 tumor samples from TCGA. The results indicated a significant elevation in SAR1A mRNA expression in tumor tissues (Figure 1C). We examined SAR1A expression in HNSCC patients at different clinical stages. Figure 1D demonstrates that the expression of SAR1A from the first to the fourth clinical stage was elevated in HNSCC. The survival analysis based on the TCGA-HNSC database https://tau.cmmt.ubc.ca/cSurvival/ (accessed on 7 October 2024) showed that HNSCC patients with high SAR1A expression exhibited lower survival probabilities (Figure 1E). The findings of the analysis suggested a correlation between high SAR1A expression and poorer survival outcomes (Figure 1E).

According to the HPA database https://www.proteinatlas.org (accessed on 6 August 2024), SAR1A is localized in the ER through immunofluorescence staining (Figure 1F).

### 3.2. The DNA Methylation Profile of SAR1A and Its Correlations with Immune Cells in HNSCC

**Figure 2:**
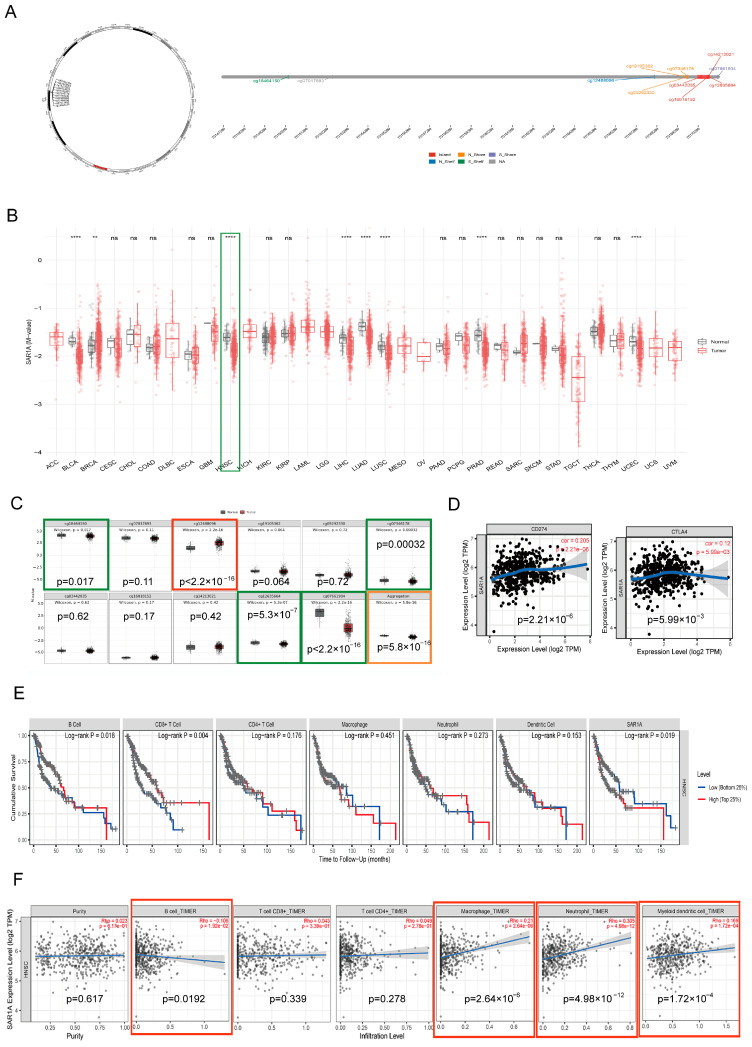
The DNA methylation profile of SAR1A and its correlations and interactions with the immune microenvironment in HNSCC: (**A**) the detailed information of 11 methylation probes for SAR1A located on chromosome 10; (**B**) methylation status of SAR1A in tumor and normal tissues in 33 different types of cancer. Normal tissues are depicted in gray, while tumor tissues are represented in red, HNSC marked with a red box; (**C**) the methylation levels of SAR1A probes and their aggregation were analyzed. The HNSCC samples exhibited a notably elevated level of methylated probes, denoted in red, alongside a decreased level of methylation, indicated in green. The methylation aggregation of SAR1A in HNSCC is prominently indicated in orange; (**D**) the correlation between CD274 (PD-L1) (*p* value: 2.21 × 10^−6^), CTLA4 (*p* value: 5.99 × 10^−3^), and SAR1A; (**E**) the cumulative survival rates in HNSCC are depicted based on the varying levels of immune cell expression and SAR1A expression; (**F**) the association between the SAR1A expression level and tumor purity, as well as immune cell infiltration level. The partial Spearman correlation was used. ** *p* < 0.01; **** *p* < 0.0001; ns, not significant.

DNA methylation constitutes an essential factor in triggering cancer. Hypermethylation status signifies the suppression of gene expression, whereas hypomethylation indicates the activation of gene expression [25]. To clarify the underlying mechanism for the upregulation of SAR1A in HNSCC, the DNA methylation status of SAR1A was investigated using the SMART software http://www.bioinfo-zs.com/smartapp/ (accessed on 6 August 2024). SAR1A is situated on chromosome 10, with a distribution of 11 methylation probes. The positions of these probes on the CpG island are illustrated in Figure 2A. The methylation status of SAR1A was investigated across 33 different types of cancer (Figure 2B). SAR1A methylation was lower in HNSCC samples than in normal samples, indicating a hypomethylation status in HNSCC patients. The methylation status of 11 probes was specifically determined (Figure 2C). Four probes demonstrated markable hypomethylation status (S_Shelf_cg1846415; N_Shore_cg07346178; Island_cg12635664; S_Shore_cg07661904), and one probe exhibited significant hypermethylation status (N_Shelf_cg12488096).

To further explore the impact of SAR1A on the immune microenvironment in HNSCC, we applied the TIMER software http://timer.cistrome.org/ (accessed on 6 August 2024) to investigate the association between SAR1A and key immune checkpoints, such as CD274 (PD-L1) and cytotoxic T-lymphocyte-associated protein 4 (CTLA4) (Figure 2D). The correlation of SAR1A with CD274 (PD-L1) was found to be 0.205 (*p* = 0.000002), and with CTLA4, it was found to be 0.12 (*p* = 0.005990). The outcome module in the TIMER2.0 software evaluated the clinical relevance of tumor immune subsets and SAR1A expression levels in HNSCC. CD8+ T cells and B cells showed an association with the cumulative survival of HNSCC patients. SAR1A expression was significantly linked to the cumulative survival of HNSCC patients (Figure 2E). We examined the correlation between SAR1A and levels of immune cell infiltration. The expression level of SAR1A was correlated with the infiltration levels of B cells, neutrophils, macrophages, and dendritic cells (Figure 2F). These findings suggest that SAR1A interacts with the immune microenvironment in HNSCC patients.

### 3.3. The Correlation Between SAR1A Expression and the Clinicopathological Characteristics of HNSCC Patients

**Figure 3:**
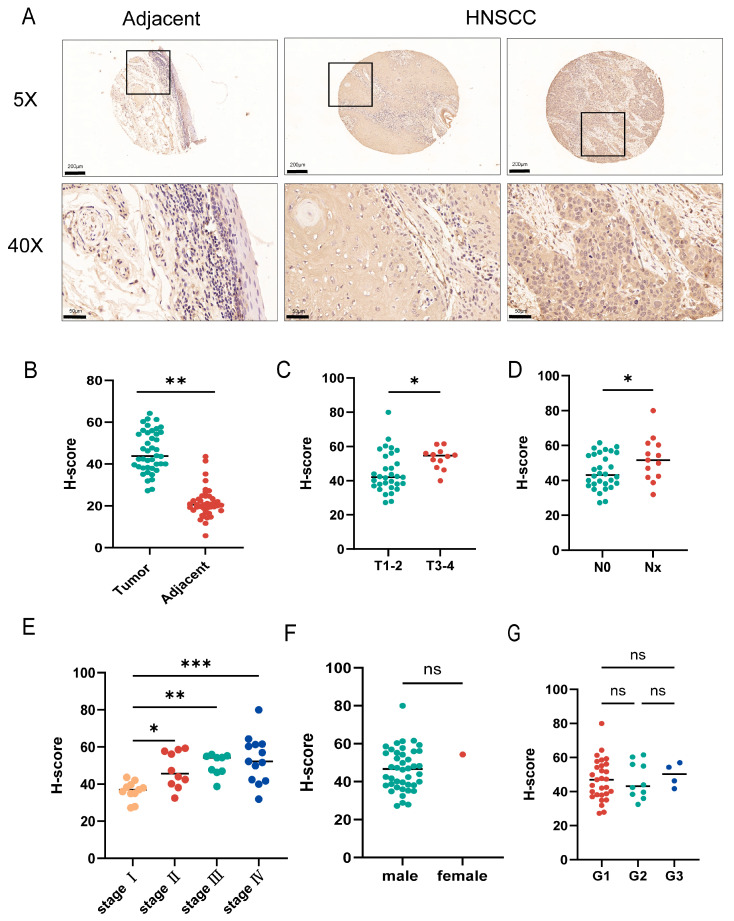
Clinical significance of SAR1A in HNSCC: (**A**) representative IHC staining images of SAR1A in a tissue microarray. Scale bars: 100 μm and 50 μm. ImageJ was used to analyze the expression levels of SAR1A using an IHC profiler according to the H-score for (**B**) HNSCC and adjacent tissues, (**C**) stages T1–2 and stages T3–4, (**D**) lymph node involvement, (**E**) clinical stages, (**F**) genders, and (**G**) tumor differentiation stages. Data are displayed as the mean  ±  SD. ns, not significant; * *p* < 0.05; ** *p* < 0.01; *** *p* <0.001.

To explore the association between SAR1A and the clinicopathological characteristics of HNSCC patients, SAR1A expression was evaluated through the IHC staining of tissue microarrays. The intensity of SAR1A staining was markedly elevated in HNSCC tissues (Figure 3A,B). The staining score of SAR1A was higher in HNSCC patients in correlation with the T stage, lymph node involvement, and the clinical stage (Figure 3C–E), but it showed no correlation with other parameters, such as gender and tumor differentiation (Figure 3F,G). Table 1 presents an overview of the general characteristics of 504 HNSCC patients stratified by SAR1A expression based on the TCGA data. Our findings indicated that there was no correlation between SAR1A expression and gender, age, or distant metastasis. High SAR1A expression was closely correlated to the T stage, N stage, histological grade, and history of alcohol use.

### 3.4. SAR1A Knockdown Inhibits HNSCC Cell Proliferation

**Figure 4:**
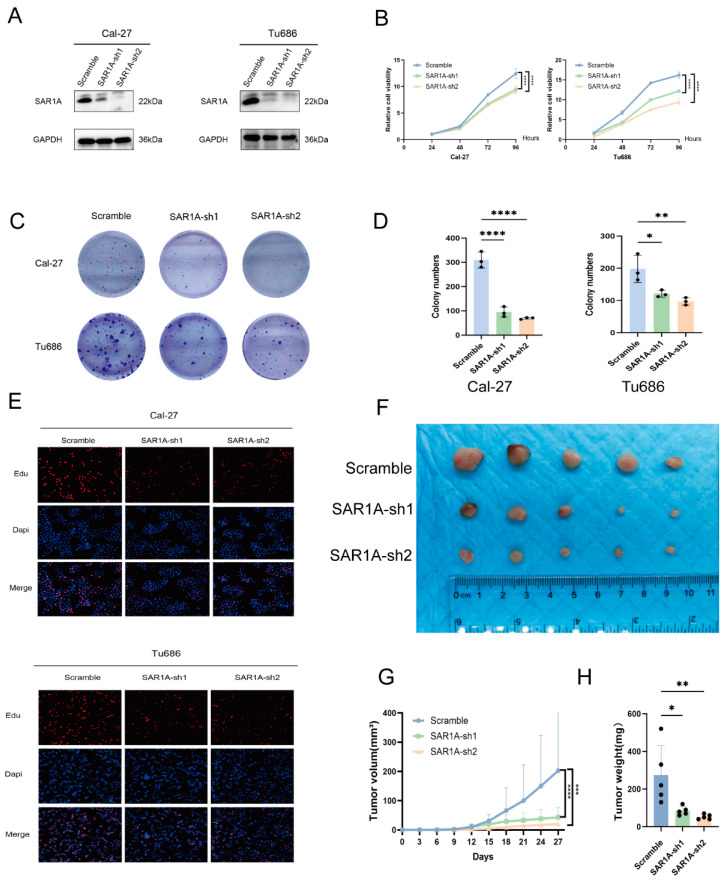
SAR1A knockdown inhibited HNSCC cell proliferation: (**A**) Western blotting was conducted to assess SAR1A expression after lentivirus-containing scramble or SAR1A-shRNA transfection; (**B**) the proliferation of cells transfected with lentivirus-containing plasmids was assessed using the CCK8 assay; (**C**) representative images depicting colony formation; (**D**) quantitative results of the colony formation analysis; (**E**) representative images of the Edu assay; (**F**) a representative image of the xenograft model in nude mice. Tumor volumes (**G**) and tumor weight (**H**) of subcutaneous tumors in nude mice. All *p* values were determined through one-way ANOVA or two-way ANOVA. Data are displayed as the mean  ±  SD. * *p* < 0.05; ** *p* < 0.01; *** *p* < 0.001; **** *p* < 0.0001.

Tumor cell proliferation is a crucial factor in carcinogenesis and progress in HNSCC. To examine the involvement of SAR1A in HNSCC proliferation, Cal-27, and Tu686 cells were transfected with lentivirus carrying either scramble or SAR1A-shRNA. The level of SAR1A protein was decreased in SAR1A-sh1 and SAR1A-sh2 groups, as observed in the Western blot analysis (Figure 4A). The CCK-8 assay demonstrated that SAR1A knockdown decreased cell viability (Figure 4B). SAR1A knockdown significantly decreased the colony formation of Cal-27 and Tu686 cells, as demonstrated by the colony formation assay (Figure 4C,D). A decrease in EdU-positive cells was observed in Cal-27 and Tu686 cells following SAR1A knockdown, as indicated by Edu assays. A xenograft assay on nude mice revealed that SAR1A knockdown reduced the growth of xenograft tumors (Figure 4F–H). These findings suggest that SAR1A knockdown effectively inhibited HNSCC cell proliferation in vivo and in vitro.

### 3.5. SAR1A Knockdown Suppresses Metastasis and Invasion in HNSCC Cells

**Figure 5:**
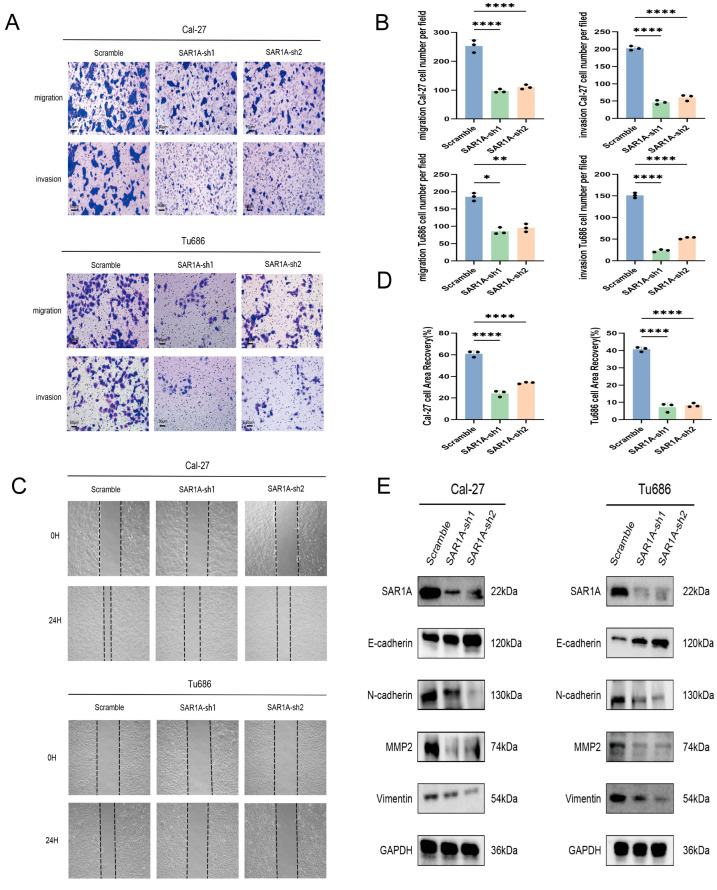
SAR1A knockdown suppressed the metastasis and invasion of HNSCC cells: (**A**) representative images of transwell assays; (**B**) the cell migration and invasion abilities were assessed with the ImageJ software; (**C**) representative images of wound-healing assays; (**D**) the migration ability was analyzed using ImageJ; (**E**) the impact of SAR1A knockdown on EMT-related markers. All *p* values were calculated through one-way ANOVA. Data are displayed as the mean  ±  SD. * *p* < 0.05; ** *p* < 0.01; **** *p* < 0.0001. The scale bar represents a distance of 50 μm.

Distant metastasis in HNSCC is correlated with an unfavorable prognosis. Transwell and wound-healing assays were conducted to investigate the involvement of SAR1A in HNSCC migration. The transwell assays determined that SAR1A knockdown suppressed the abilities of migration and invasion in Caal-27 and Tu686 cells (Figure 5A,B). The wound-healing assays further confirmed the inhibited migration capabilities of HNSCC cells following SAR1A knockdown (Figure 5C,D). The process of EMT is crucial for the regulation of cancer metastasis and has been demonstrated to significantly enhance invasive potential. We conducted Western blotting to investigate the levels of EMT markers in SAR1A knockdown and scramble HNSCC cells. The expression level of E-cadherin was elevated. Additionally, the level of N-cadherin, Vimentin, Snail, and MMP-2 were reduced in SAR1A knockdown cells, as determined through Western blotting (Figure 5E). The findings suggest that SAR1A knockdown inhibits the migratory and invasive capabilities of HNSCC cells.

### 3.6. SAR1A Affects the PI3k/Akt/mTOR Signaling Pathway in HNSCC Cells

**Figure 6:**
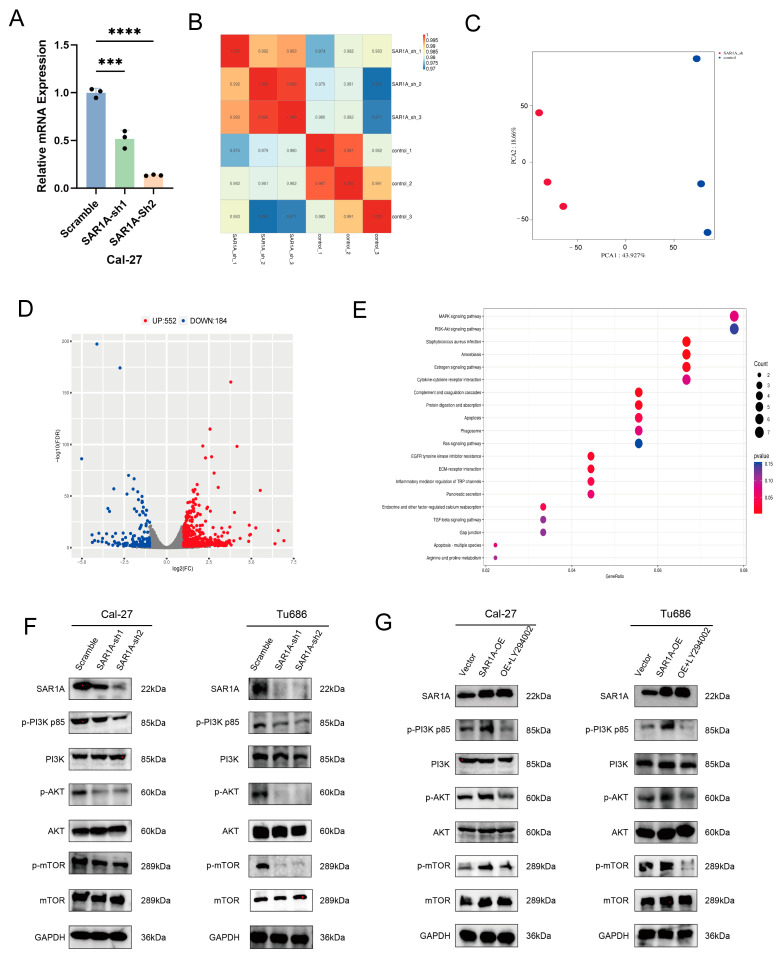
RNA sequencing after downregulating SAR1A and Western blotting for the PI3K/AKT/mTOR pathway with or without LY294002: (**A**) the effect of SAR1A knockdown in Cal-27 cells was confirmed using qRT-PCR; (**B**) a correlation heatmap of the gene expression levels among the samples. The samples were evaluated for correlations using the Pearson correlation coefficient. Red indicates a strong correlation, blue indicates a weak correlation; (**C**) a two-dimensional PCA analysis was conducted, with each point representing a single sample. The distance between the two samples indicates the extent of the difference in their distribution patterns. Control samples are depicted in blue, while knockdown samples are represented in red; (**D**) a volcano plot after SAR1A knockdown for the 736 DEGs in Cal-27 cells. Red shows upregulated DEGs, and blue indicates downregulated DEGs. Gray indicates genes that showed no significant differences; (**E**) a bubble plot illustrating the results of KEGG pathway analysis, depicting the functional changes in genes following SAR1A knockdown; (**F**) Western blot analysis of the impacts of SAR1A knockdown on the PAM pathway in HNSCC cells; (**G**) Western blot assays were conducted to examine the impacts of overexpression of SAR1A and treatment with LY290042 (25 μM) for 24 h on the PAM pathway. All *p* values were determined through one-way ANOVA. Data are displayed as the mean  ±  SD. *** *p* < 0.001; **** *p* < 0.0001.

To further elucidate the mechanism of SAR1A in HNSCC carcinogenesis, we confirmed a reduction in mRNA levels of SAR1A in SAR1A-sh1 and SAR1A-sh2 HNSCC cells (Figure 6A), which was followed by transcriptome sequencing of Cal-27 cells from SAR1A knockdown and control groups. The experiment’s reliability and the rationality of sample selection were assessed based on the correlation of gene expression between samples. A correlation coefficient closer to 1 indicates greater similarity between the samples. The control group and the SAR1A-sh group showed high within-group sample correlation, indicating good reproducibility. However, the correlation between samples in the control and SAR1A groups decreased, which may suggest differences in gene expression patterns between the two groups (Figure 6B). The Q20 value (the percentage of bases with sequencing errors below 1%) for all samples ranged from 97.31% to 97.59%, while the Q30 value (the percentage of bases with sequencing errors below 0.1%) ranged from 91.86% to 92.49%. Furthermore, 99.33–99.45% of the samples were successfully mapped to the reference genome, indicating reliable sequencing results (Table 2). Figure 6C depicts a 2D plot of the PCA analysis, showing distinct separation between the control and KD groups, as well as close proximity among samples within each group, indicating high repeatability. Based on the screening criteria for DEGs, we identified a total of 736 DEGs, consisting of 184 downregulated DEGs and 552 upregulated DEGs (Figure 6D). Through KEGG analysis and pathway enrichment, we identified that the predominant changes in gene transcriptome expression occurred within the PAM pathway (Figure 6E). Western blot analysis demonstrated that the knockdown of SAR1A significantly suppressed the protein levels of phospho-PI3K, phospho-AKT, and phospho-mTOR. However, no alterations were observed in the levels of total PI3K, AKT, and mTOR (Figure 6F). To validate the impact of the PAM pathway on HNSCC cells, we treated cells overexpressing SAR1A with LY294002 [26] for 24 h. The findings revealed that the inhibition of PI3K/AKT led to a reduction in the levels of phosphorylated PI3K, AKT, and mTOR proteins, while no alterations were observed in the level of total PI3K, AKT, and mTOR (Figure 6G). These results demonstrate that SAR1A influences the PAM pathway in HNSCC cells.

### 3.7. SAR1A Regulates HNSCC Proliferation via the PI3K/AKT/mTOR Pathway

**Figure 7:**
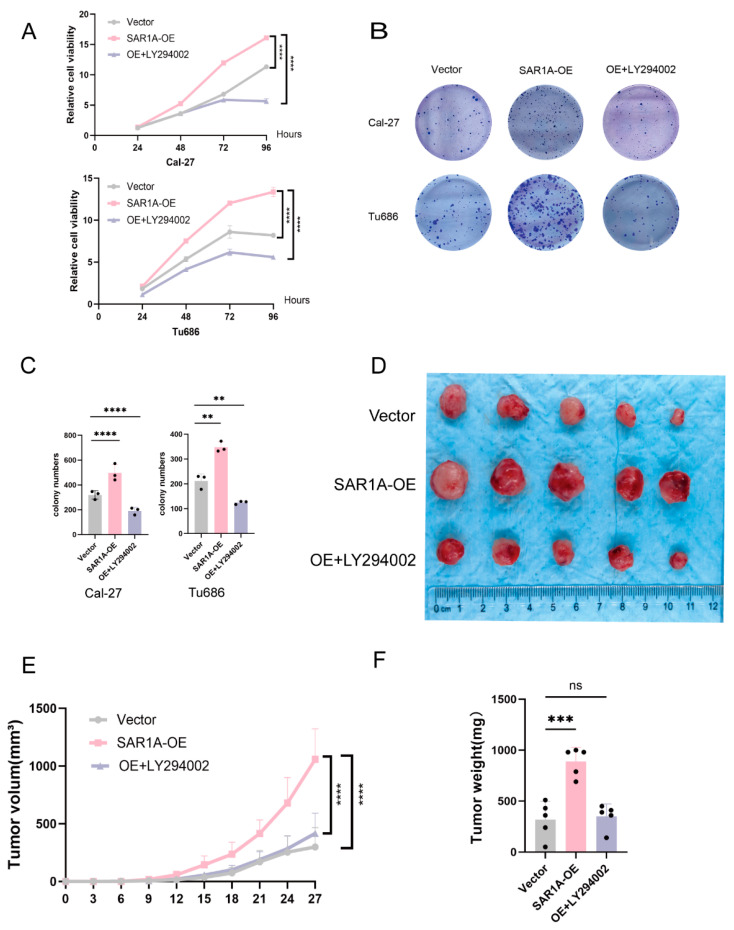
SAR1A regulated HNSCC proliferation via the PI3K/AKT/mTOR pathway. The capacities of proliferation were determined using (**A**) the CCK8 assay and (**B**) colony formation assays; (**C**) analysis of the formation of colonies using ImageJ; (**D**) a representative image of the xenograft model in nude mice. Tumor volumes (**E**) and tumor weight (**F**) of subcutaneous tumors in nude mice. All *p* values were determined through one-way ANOVA or two-way ANOVA. Data are displayed as the mean  ±  SD. ns, not significant; ** *p* < 0.01; *** *p* < 0.001; **** *p* < 0.0001.

To further investigate the impact of SAR1A regulation on the PAM signaling pathway, SAR1A-overexpressing Cal-27 and Tu686 cells were subjected to treatment with LY294002 (25 μM for 24 h). CCK8 assays indicated that the SAR1A-OE groups exhibited significantly higher proliferation capacity than that of the EV-phage groups. However, treatment with LY294002 (25 μM for 24 h) resulted in a marked reduction in the proliferation of the SAR1A-overexpressing groups (Figure 7A). Furthermore, the number of clones in the SAR1A-overexpressing groups exhibited a significant increase compared with both the SAR1A-EV groups and the SAR1A-overexpressing groups treated with LY294002. The colony formation assay further validated that the PI3K/AKT inhibitor significantly reduced HNSCC cell proliferation driven by elevated expression of SAR1A (Figure 7B). To investigate the impact of SAR1A on xenograft growth through the PI3K/AKT/mTOR axis, Cal-27 cells were subcutaneously implanted into nude mice. The overexpression of SAR1A promoted xenograft growth, whereas treatment with the PI3K/AKT inhibitor LY294002 resulted in inhibition (Figure 7D). Furthermore, the calculation of xenograft volumes (Figure 7E) and weights (Figure 7F) revealed a decrease in the SAR1A-OE+LY294002 group compared to the SAR1A-OE group. These results indicate that SAR1A overexpression promotes the proliferation of HNSCC cells via the PAM signaling pathway, whereas the suppression of the PI3K/AKT pathway reverses these effects on HNSCC cell proliferation.

### 3.8. SAR1A Regulates the EMT of HNSCC Cells via the PI3K/AKT/mTOR Pathway

**Figure 8:**
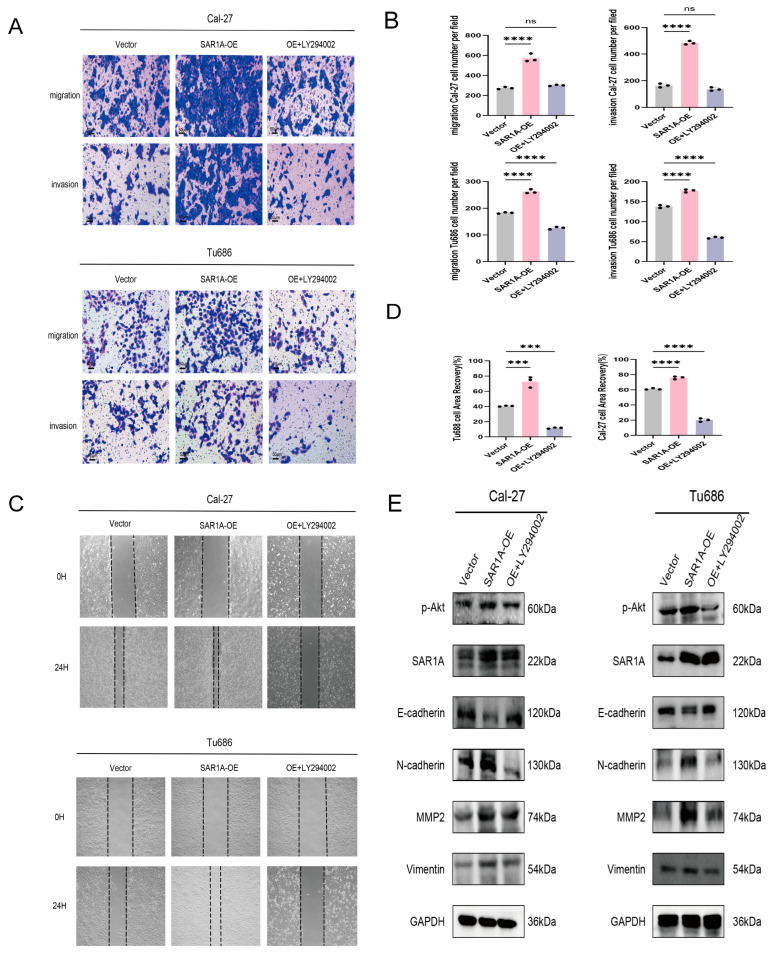
SAR1A regulates the EMT in HNSCC cells via the PI3K/AKT/mTOR pathway: (**A**,**B**) transwell and wound-healing assays (**C**,**D**) were conducted in SAR1A-overexpressing cells with or without treatment with LY294002; (**E**) Western blot were conducted to assess the impact of LY294002 on EMT-related markers and *p*-AKT in SAR1A-overexpressing cells. Data are displayed as the mean  ±  SD. All *p* values were determined through one-way ANOVA. ns, not significant; *** *p* < 0.001; **** *p* < 0.0001. The scale bar represents a distance of 50 μm.

To investigate the impact of SAR1A’s promotion of the EMT through the PAM pathway, cells overexpressing SAR1A were treated with LY294002. Transwell assays demonstrated increased migratory and invasive capabilities in SAR1A-overexpressing cells, which were attenuated by LY294002 treatment (Figure 8A,B). Consistent results were observed in wound-healing assays, where SAR1A overexpression expanded the cell migration area, while the LY294002 treatment reduced it (Figure 8C,D). Western blot analysis revealed a reduction in E-cadherin levels and an elevation in N-cadherin, MMP-2, and Vimentin levels after SAR1A overexpression. Treatment with LY294002 led to an elevation in E-cadherin levels and a reduction in N-cadherin, MMP-2, and Vimentin expression levels compared with SAR1A overexpression (Figure 8E). These findings collectively indicate that SAR1A promotes the EMT in HNSCC cells via the PAM pathway. The inhibition of PI3K/AKT reverses the EMT process induced by SAR1A overexpression in HNSCC cells.

### 3.9. SAR1A Enhances In Vivo Metastasis of HNSCC Cells

**Figure 9:**
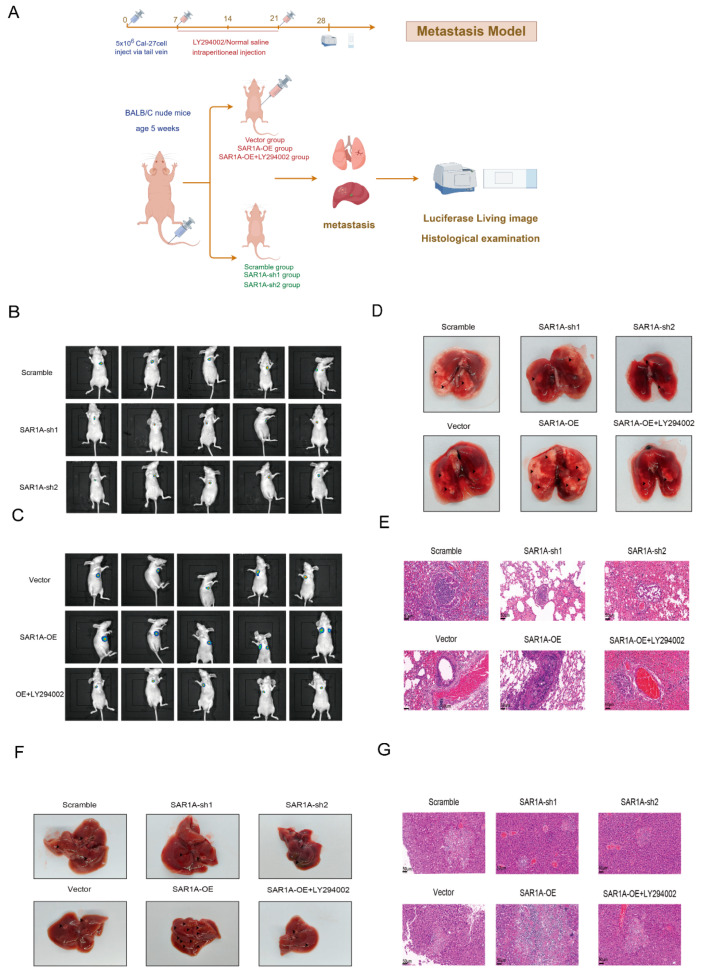
SAR1A enhances the metastasis of HNSCC cells in vivo: (**A**) schematic representation of metastatic models in nude mice, where Cal-27 cells were administered through injection via the tail vein; (**B**,**C**) representative bioluminescent images of nude mice; representative images of lungs (**D**) and livers (**F**). The presence of metastatic nodules is indicated by black arrows. The lungs (**E**) and livers (**G**) were observed using H&E staining. The scale bar represents a distance of 50 μm.

To validate the metastatic potential of SAR1A in vivo, we established nude mouse lung metastasis models through the intravenous injection of 5 × 106 Cal-27-Luc cells that were stably transfected with specific plasmids (Figure 9A). Intravenous administration of SAR1A-sh1 and SAR1A-sh2 cells led to decreased accumulation of the fluorescence signal (Figure 9B). Conversely, SAR1A-OE groups exhibited higher levels of fluorescence signal accumulation. Following the intraperitoneal injection of LY294002, there was a reduction in the accumulation of the fluorescence signal (Figure 9C). To validate this hypothesis, lung and liver tissues were extracted for macroscopic observation (Figure 9D,F) and HE staining (Figure 9E,G) to identify metastatic nodules. Compared with the SAR1A-NC group, both SAR1A-sh1 and SAR1A-sh2 showed fewer metastatic nodules in the lungs and livers, while the SAR1A-OE group displayed an increased number of these nodules compared with the SAR1A-EV group. Treatment with LY294002 resulted in a decrease in metastatic nodules in the lungs and livers. Our findings suggest that SAR1A enhances the in vivo metastasis of HNSCC cells.

## 4. Discussion

HNSCC is a significant health concern, with over fifty percent of patients diagnosed at an advanced stage [27]. The prognosis for locoregionally advanced HNSCC remains poor, with the five-year OS rates ranging from 30% to 50% following surgical intervention [28]. Studying new biomarkers and advanced therapeutic approaches is crucial for enhancing the prognosis of HNSCC.

In our study, we identified 38 DEGs from the TCGA database and HDAb website. Through univariate and multivariate regression analyses, we established a correlation between the risk ratio of these DEGs and patient prognosis. Notably, SAR1A emerged as a key gene, exhibiting the highest HR among the identified DEGs. Kaplan–Meier survival analysis further demonstrated that elevated SAR1A levels were significantly correlated with poor OS in HNSCC patients.

SAR1A may exert varying effects in different types of tumors. SAR1A is upregulated in colon cancer [29] and osteosarcoma [20], where it promotes osteosarcoma cell metastasis through the RhoA/YAP pathway, ER stress, and autophagy. In contrast, SAR1A functions as a tumor suppressor in lung cancer [16], liver cancer [21], and glioblastoma [8]. The deficiency of both SAR1A and SAR1B promotes the growth of orthotopically transplanted lung tumors in mice [16]. In hepatocellular carcinoma (HCC) cells, SAR1A interacts with TMEM176A to inhibit ERK signaling, thereby suppressing HCC cell growth [21]. Additionally, SAR1A interacts with Cytochrome P450 (CYP) 17A1 to regulate protein processing in the ER, contributing to the survival of glioblastoma cells [8]. Our analysis of TCGA data and clinical samples revealed that SAR1A expression was increased in HNSCC samples, and high expression was linked to poor prognosis. This suggests a potential carcinogenic role for SAR1A in HNSCC. Our hypothesis was confirmed through in vitro experiments, such as a cck-8 assay, Edu assay, colony formation assay, wound-healing assay, and transwell migration assay, as well as in vivo experiments, such as those on xenograft models and metastasis models.

To investigate the potential mechanism by which SAR1A influences the proliferation and migration of HNSCC cells, we conducted high-throughput transcriptome sequencing analysis on SAR1A knockdown cells and a control group. The results revealed significant changes in the PAM pathway. We validated the RNA-seq results through experiments such as Western blotting.

The PAM pathway plays a crucial role in mediating cell growth, differentiation, proliferation, apoptosis, and other aspects [30]. It is considered one of the most critical intracellular signaling pathways for maintaining the basic functions of cells [31]. The PAM pathway plays a central role in carcinogenesis and progression and is one of the most frequently activated signaling pathways [32]. PAM signaling is active in over 90% of HNSCC [33]. Heat shock protein 90 beta family member 1 (HSP90B1) modulates autophagy through the PAM pathway, facilitating proliferation, migration, and invasion, and inhibiting the apoptosis of HNSCC [34]. Integrin beta 2 (ITGB2) plays a pivotal role in promoting OSCC proliferation in CAFs by enhancing the glycolytic activity of cancer-associated fibroblasts (CAFs) via the PAM pathway [35]. The Na^+^-coupled bicarbonate transporter, SLC4A7, promotes EMT and HNSCC metastasis through the PAM signaling pathway [36]. Chemokine receptor 7 (CCR7) activates NF-κB via PAM to promote HNSCC cell invasion and survival [37]. SOAT1 facilitates OSCC progression by upregulating the SREBP1-regulated adipogenic pathway and activating the PAM pathway [38]. NF-κB facilitates the proliferation, survival, migration, inflammation, and angiogenesis of tumor cells [39]. LY-294002 demonstrated the ability to attenuate both constitutive and inducible NF-κB activity in HNSCC [40]. The mutual crosstalk between the PAM pathway and the NF-κB pathway needs to be further investigated in HNSCC. The PAM pathway is concomitantly activated with the Ras/Raf (MAPK kinase kinase)/MAPK pathway downstream from EGFR [41]. The MAPK signaling pathway was also identified in the results of a KEGG pathway analysis (Figure 6E). It is noteworthy that ERK/MAPK, JNK/MAPK, and P38/MAPK demonstrate either oncogenic or tumor-suppressing effects in different tissues or through crosstalk among various signaling pathways [42]. Further investigation is warranted to elucidate the association between SAR1A and the MAPK pathway.

Despite the promising findings, this study has several constraints. The mechanisms underlying SAR1A’s impact on HNSCC progression remain incompletely elucidated, and further investigation is needed to understand the crosstalk between SAR1A and other signaling pathways. Additionally, potential biases introduced by reliance on bioinformatics analyses must be addressed through additional experimental validation. Furthermore, there is currently a lack of research specifically focused on inhibitors targeting SAR1A. The development of SAR1A inhibitors and their combination with radiation therapy, chemotherapy, or PAM pathway inhibitors may offer a novel approach to overcoming resistance in the treatment of HNSCC in the future. The advantages of this study lie in the comprehensive analysis of DEGs, the correlation of SAR1A expression with clinical outcomes, and the use of a combination of in vitro and in vivo experiments to support our hypotheses. The identification of SAR1A as a promising biomarker provides a potential therapeutic target for future interventions in HNSCC. Future work should focus on elucidating the precise mechanisms through which SAR1A regulates the PAM pathway and identifying its interacting molecules. Furthermore, exploring the potential of SAR1A’s inhibitor as a therapeutic target in combination with existing treatment modalities could lead to more effective strategies for treating HNSCC. Investigating the crosstalk between SAR1A and other signaling pathways, such as the NF-κB and MAPK pathways, could also provide insight into the complex network of interactions involved in HNSCC progression.

## 5. Conclusions

**Figure 10:**
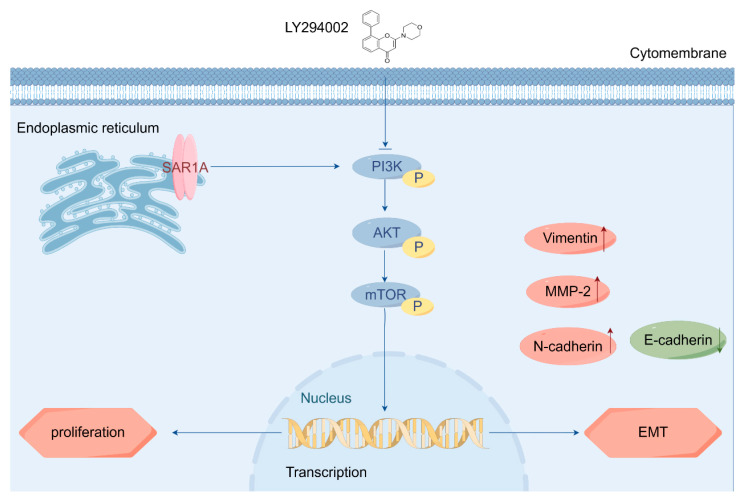
Schematic representation of the potential mechanism of SAR1A in HNSCC. SAR1A facilitates HNSCC proliferation and the EMT through the PI3K/AKT/mTOR pathway. EMT: epithelial–mesenchymal transition. An upward arrow indicates an increase, while a downward arrow signifies a decrease.

In conclusion, our study highlights the significance of SAR1A as a potential biomarker and its underlying mechanisms (Figure 10) in HNSCC. Key findings include the following:

- Notably, 38 DEGs were identified, with SAR1A showing the highest HR value of prognosis;
- High levels of SAR1A exhibited a correlation with poor OS in HNSCC patients;
- In vitro and in vivo experiments demonstrated that SAR1A promotes cell proliferation, migration, and invasion;
- Transcriptome analysis indicated that SAR1A facilitates HNSCC cell proliferation and migration through the PAM pathway;
- The PI3K/AKT inhibitor LY294002 reversed the enhanced proliferation, migration, and invasion abilities of HNSCC cells induced by SAR1A overexpression.

## Figures and Tables

**Table 1:** SAR1A expression and clinical characteristics of HNSCC based on the TCGA database.

Characteristics	Number of Patients	Expression of SAR1A		*p* Value
		Low, *n* =	High, *n* =	
Gender				
Male	370	179 (35.5%)	191 (37.9%)	0.226
Female	134	73 (14.5%)	61 (12.1%)	
Age (year)				
≤60	248	121 (24%)	127 (25.2%)	0.562
>60	256	131 (26%)	125 (24.8%)	
T stage				
T1	45	30 (6.7%)	15 (3.3%)	
T2	135	73 (16.3%)	62 (13.8%)	0.048
T3	96	46 (10.3%)	50 (11.2%)	
T4	172	77 (17.2%)	95 (21.2%)	
N stage				
N0	171	101 (24.6%)	70 (17%)	
N1	66	31 (7.5%)	35 (8.5%)	0.008 **
N2 and N3	174	74 (18%)	100 (24.3%)	
Distant metastasis				
M0	188	100 (52.9%)	88 (46.6%)	0.471
M1	1	0 (0%)	1 (0.5%)	
Histological grade				
G1	62	40 (8.3%)	22 (4.5%)	
G2	301	150 (31%)	151 (31.2%)	0.012 *
G3	119	49 (10.1%)	70 (14.5%)	
G4	2	2 (0.4%)	0 (0%)	
Alcohol history				
No	159	99 (20.1%)	60 (12.2%)	<0.001 **
Yes	334	145 (29.4%)	189 (38.3%)	
Smoker				
No	123	64 (13%)	59 (9.9%)	0.088
Yes	381	181 (36.6%)	200 (40.5%)	

* *p* < 0.05; ** *p* < 0.01.

**Table 2:** Results of the quality control analysis.

Sample	Raw Reads	Raw Bases	Clean Reads	Clean Bases	Clean Ratio	Q20	Q30	GC
SAR1A_sh_1	77,276,070	11,591,410,500	76,758,606	11,450,860,970	99.33%	97.52%	92.91%	46.96%
SAR1A_sh_2	51,500,038	7,725,005,700	51,194,288	7,638,078,631	99.41%	97.52%	92.90%	46.81%
SAR1A_sh_3	55,816,568	8,372,485,200	55,482,230	8,288,240,595	99.40%	97.46%	92.77%	46.89%
control_1	41,472,682	6,220,902,300	41,245,862	6,167,283,950	99.45%	97.59%	92.99%	46.37%
control_2	39,068,586	5,860,287,900	38,815,060	5,805,767,053	99.35%	97.31%	92.44%	46.34%
control_3	38,962,370	5,844,355,500	38,717,308	5,767,133,804	99.37%	97.53%	92.91%	46.87%

## Data Availability

The datasets used during the current study are available from the corresponding author upon reasonable request.

## Supplementary Materials

The following supporting information can be downloaded at: https://www.mdpi.com/article/10.3390/biomedicines12112477/s1. Supplementary Figure S1. The heatmap depicting the 38 differentially expressed ARGs in HNSCC and normal tissues. Supplementary Figure S2. Identification of prognosis-associated ARGs using the Univariate Cox analysis. Supplementary Figure S3. (A) The heatmap depicting the gene expressions associated with high and low risk in HNSCC; (B) The distribution of risk scores, patient survival times, and HNSCC status; (C) Kaplan-Meier curves depicting the survival outcomes of the high- and low-risk groups; (D) Forest plots depicting the results of univariate and multivariate cox regression analyses; (E) The ROC curves used to evaluate the efficacy of Cox regression. Supplementary Table S1. List of primers used for qRT-PCR analysis.

## Abbreviations

ACC	Adrenocortical carcinoma
BLCA	Bladder urothelial carcinoma
BRCA	Breast invasive carcinoma
CESC	Cervical squamous cell carcinoma
CHOL	Cholangiocarcinoma
COAD	Colon adenocarcinoma
DLBC	Diffuse large B-cell lymphoma
ESCA	Esophageal carcinoma
GBM	Glioblastoma multiforme
HNSC	Head and neck cancer
KICH	Kidney chromophobe
KIRC	Kidney renal clear cell carcinoma
KIRP	Kidney renal papillary cell carcinoma
LAML	Acute myeloid leukemia
LIHC	Liver hepatocellular carcinoma
LUAD	Lung adenocarcinoma
LUSC	Lung squamous cell carcinoma
MESO	Mesothelioma
OV	Ovarian serous cystadenocarcinoma
PAAD	Pancreatic adenocarcinoma
PCPG	Pheochromocytoma and paraganglioma
READ	Rectum adenocarcinoma
PRAD	Prostate adenocarcinoma
SARC	Sarcoma
SKCM	Skin cutaneous melanoma
STAD	Stomach adenocarcinoma
TGCT	Testicular germ cell tumors
THC	Thyroid carcinoma
THYM	Thymoma
UCEC	Uterine corpus endometrial carcinoma
UCS	Uterine carcinosarcoma
UVM	Uveal melanoma
