# Supplementary material for: SAR1A Induces Cell Growth and Epithelial–Mesenchymal Transition Through the PI3K/AKT/mTOR Pathway in Head and Neck Squamous Cell Carcinoma: An In Vitro and In Vivo Study
Source: Biomedicines. 2024 Oct 28;12(11):2477. doi: 10.3390/biomedicines12112477 (PMC11591717; doi:10.3390/biomedicines12112477)
Supplement: Supplementary file 1 [file biomedicines-12-02477-s001.zip › Supplementary Figure.pdf]

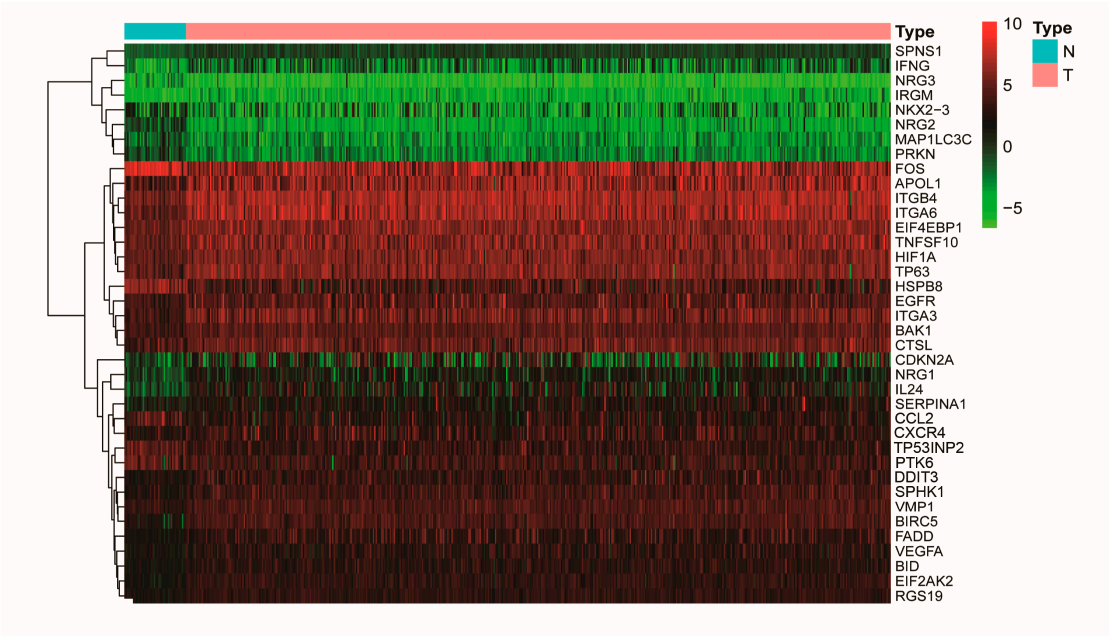

Supplementary Figure S1.The heatmap depicting the 38 differentially expressed ARGs in HNSCC and normal tissues.

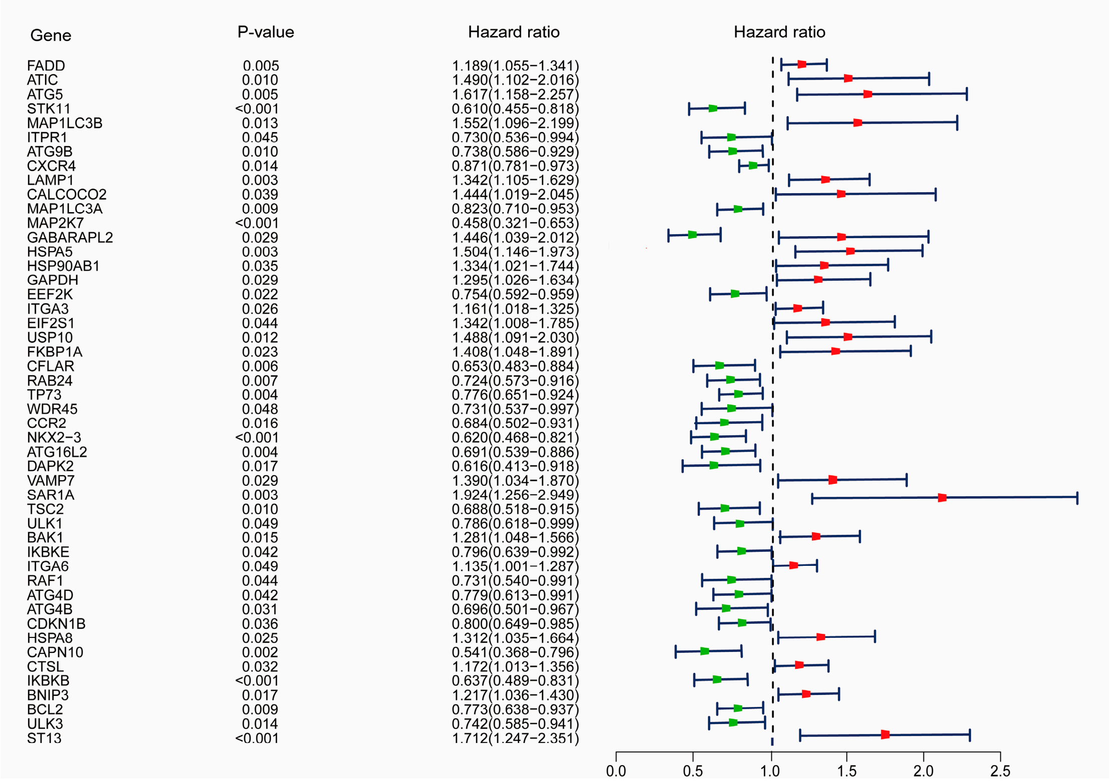

Supplementary Figure S2.Identification of prognosis-associated ARGs using the Univariate Cox analysis.

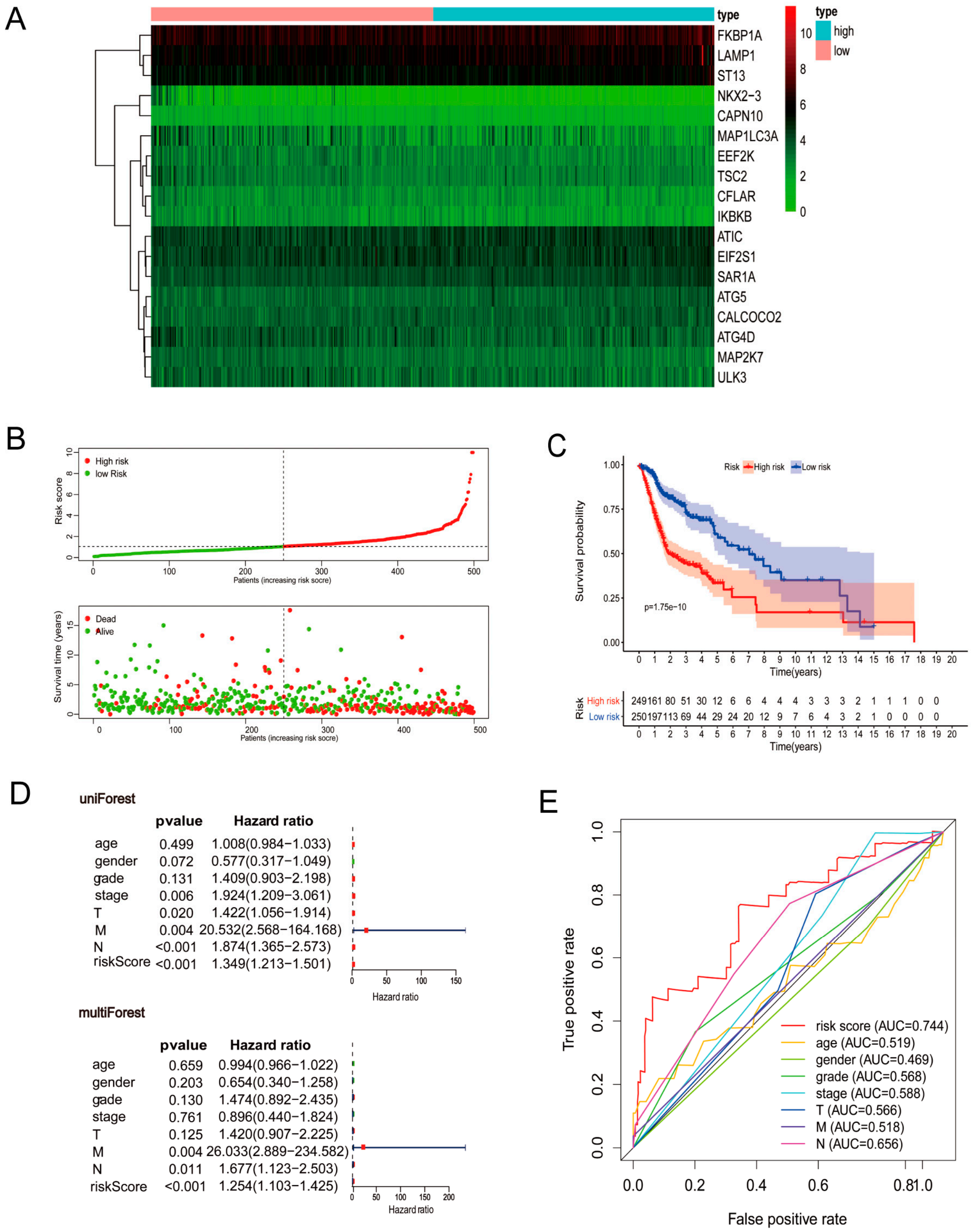

Supplementary Figure S3.(**a**)The heatmap depicting the gene expressions associated with high and low risk in HNSCC;(**b**)The distribution of risk scores, patient survival times, and HNSCC status;(**c**)Kaplan-Meier curves depicting the survival outcomes of the high- and low-risk groups;(**d**)Forest plots depicting the results of univariate and multivariate cox regression analyses;(**e**)The ROC curves used to evaluate the efficacy of Cox regression.
