# Supplementary material for: SAR1A Induces Cell Growth and Epithelial–Mesenchymal Transition Through the PI3K/AKT/mTOR Pathway in Head and Neck Squamous Cell Carcinoma: An In Vitro and In Vivo Study
Source: Biomedicines. 2024 Oct 28;12(11):2477. doi: 10.3390/biomedicines12112477 (PMC11591717; doi:10.3390/biomedicines12112477)
Supplement: Supplementary file 1 [file biomedicines-12-02477-s001.zip › Supplementary Table S1. List of primers used for qRT-PCR analysis.pdf]

Supplementary Table S1. List of primers used for qRT-PCR analysis

Genes	Primer	Primer sequence(5′–3′)
GAPDH	Forward primer	ACAACTTTGGTATCGTGGAAGG
GAPDH	Reverse primer	ACAACTTTGGTATCGTGGAAGG
SAR1A	Forward primer	ATAATGCAGGCAAAACCACTCT
SAR1A	Reverse primer	TGATGTCGGATGTAGTGTTGGAA
